# Supplementary material for: Engineering T cells to enhance 3D migration through structurally and mechanically complex tumor microenvironments
Source: Nat Commun. 2021 May 14;12:2815. doi: 10.1038/s41467-021-22985-5 (PMC8121808; doi:10.1038/s41467-021-22985-5)
Supplement: Supplementary file 1 — Supplementary Information [file 41467_2021_22985_MOESM1_ESM.pdf]

Supplementary Information

Engineering T cells to enhance 3D migration through structurally and mechanically complex tumor microenvironments

Erdem D. Tabdanov [1,2,3*], Nelson J. Rodríguez-Merced [1,2*], Alexander X. Cartagena-Rivera [4], Vikram V. Puram [1,2,5], Mackenzie K. Callaway [1,2], Ethan A. Ensminger [1,2], Emily J. Pomeroy [6,7,8], Kenta Yamamoto [6,7,8], Walker S. Lahr [6,7], Beau R. Webber [6,7,8,9], Branden S. Moriarity [6,7,8,9], Alexander S. Zhovmer [10], and Paolo P. Provenzano [1,2,6,9,11]

Affiliations:

[1] Department of Biomedical Engineering, University of Minnesota, Minneapolis, Minnesota
[2] University of Minnesota Physical Sciences in Oncology Center, Minneapolis, Minnesota
[3] Department of Pharmacology, Penn State College of Medicine, Hershey, Pennsylvania
[4] Section on Mechanobiology, National Institute of Biomedical Imaging and Bioengineering, National Institutes of Health, Bethesda, Maryland
[5] University of Minnesota Medical School, Minneapolis, Minnesota
[6] Masonic Cancer Center, University of Minnesota, Minneapolis, Minnesota
[7] Department of Pediatrics, University of Minnesota
[8] Center for Genome Engineering, University of Minnesota, Minneapolis, MN, USA
[9] Stem Cell Institute, University of Minnesota, Minneapolis, Minnesota
[10] National Heart, Lung, and Blood Institute, National Institutes of Health, Bethesda, Maryland
[11] Institute for Engineering in Medicine, University of Minnesota, Minneapolis, Minnesota

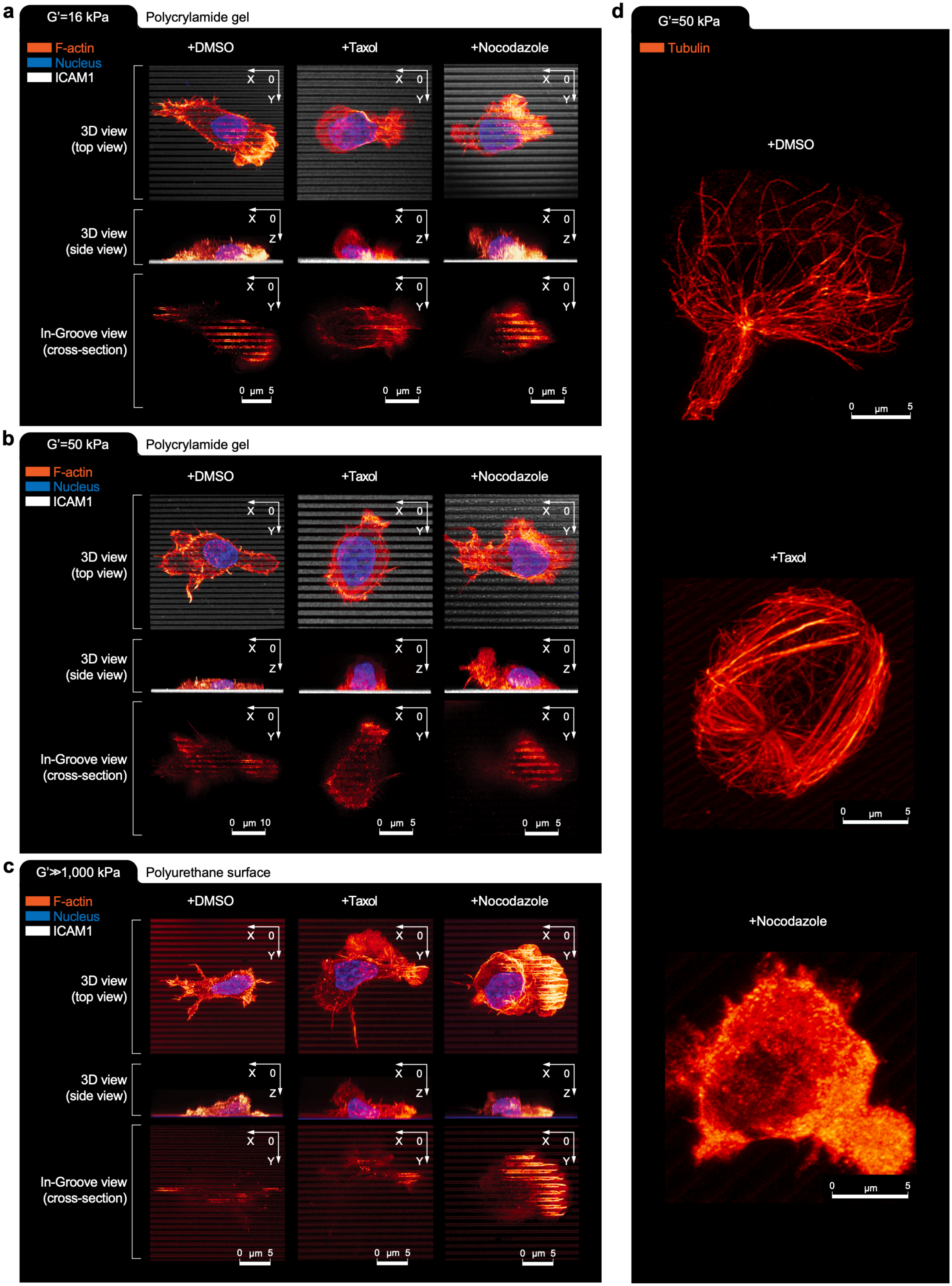

a
G'=16 kPa
Polycrylamide gel
F-actin
Nucleus
ICAM1
+DMSO
+Taxol
+Nocodazole
3D view
(top view)
3D view
(side view)
In-Groove view
(cross-section)
X 0 Y
X 0 Z
0 µm 5
b
G'=50 kPa
Polycrylamide gel
F-actin
Nucleus
ICAM1
+DMSO
+Taxol
+Nocodazole
3D view
(top view)
3D view
(side view)
In-Groove view
(cross-section)
0 µm 10
0 µm 5
c
G'≫1,000 kPa
Polyurethane surface
F-actin
Nucleus
ICAM1
+DMSO
+Taxol
+Nocodazole
3D view
(top view)
3D view
(side view)
In-Groove view
(cross-section)
0 µm 5
d
G'=50 kPa
Tubulin
+DMSO
+Taxol
+Nocodazole
0 µm 5

Supplementary Figure 1. Super-resolution STED 3D microscopy reconstructions of the invasiveness of human cells on nanotopographies of distinct stiffness (related to Figure 1). (a-c, each panel) hCD4+ T cell “in-groove” invasiveness: Left - control conditions (+DMSO), Middle - under microtubules-stabilizing Taxol treatment (+Taxol), Right - under microtubules-destabilization conditions (+Nocodazole). **(a)** G’=16 kPa, **(b)** G’=50 kPa and **(c)** G’≫1,000 kPa. **(a-c, each panel)** Top - 3D reconstruction view (Top view); Middle - 3D X0Z plane (Side view); Bottom - “in-groove” X0Y plane view (Cross-section). Note the decrease in T cell “in-groove” invasiveness in control conditions (+DMSO) as the rigidity G’ increases, while Taxol treatment universally decreases “in-groove” invasiveness and Nocodazole treatment universally increases T cell “in-groove” invasiveness. Scale: nanogroove/nanoridge: 800/800 nm. Colors: red - F-actin, white - ICAM1 and blue - nuclei. **(d)** STED imaging of Tubulin (red) showing that Taxol treatment results in robust microtubule filament structures, consistent with the MT stabilizing mechanism of Taxol, while Nocodazole treatment results in loss of microtubule filaments with only diffuse monomeric tubulin signal remaining.

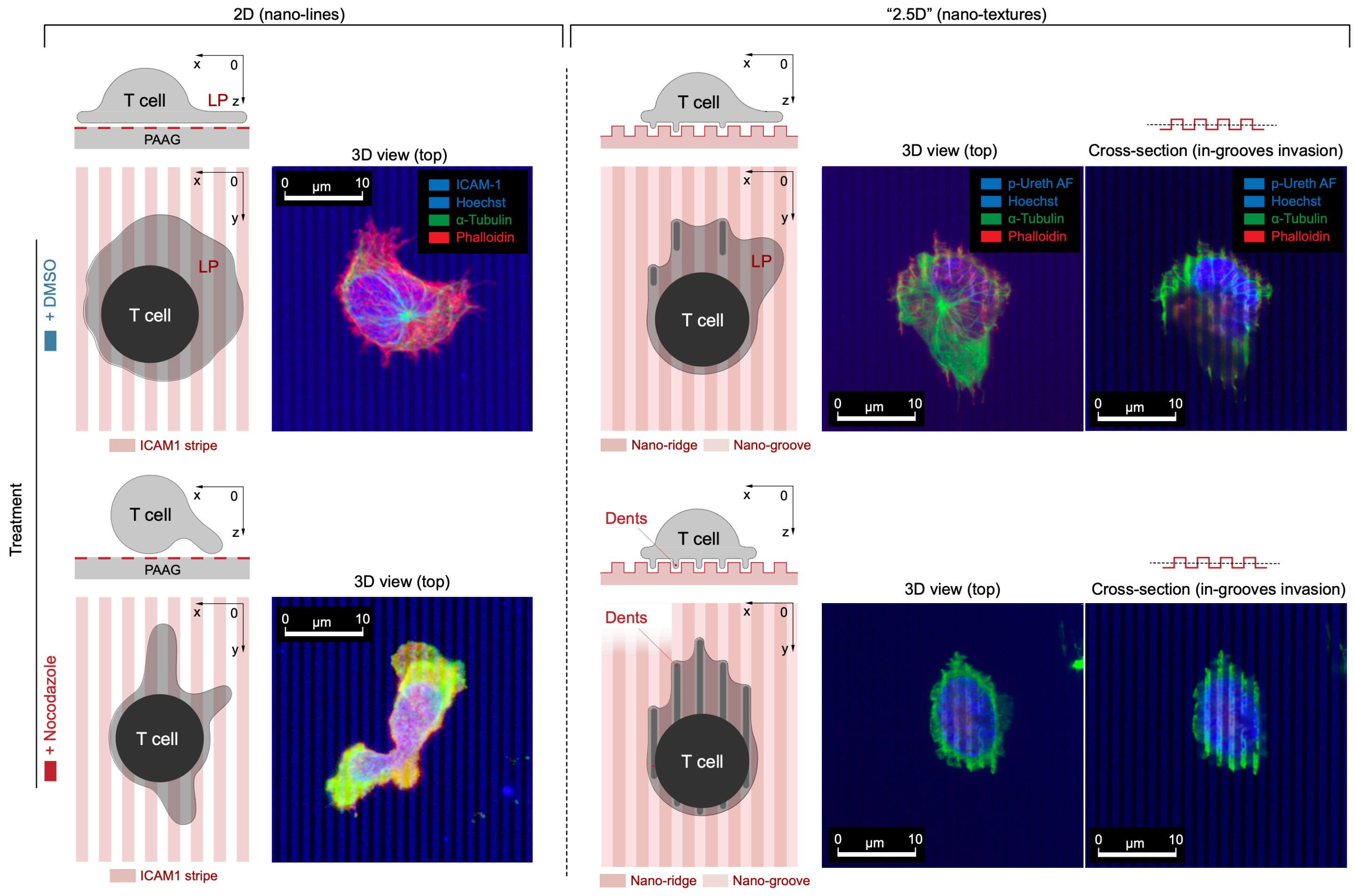

Supplementary Figure 2. T cell morphology, spreading, and "in-groove" invasiveness under various treatments. Left - Flat 2D surface with ICAM1 nanolines (G'=50 kPA). Right - Nanotextured "2.5D" ICAM1 surface (G'=50 kPa). hCD4+ T cells develop a flat spreading architecture on both nanolines and nanotextures in the control (+DMSO) conditions, combined with the partial "in-groove" invasiveness (right). Nocodazole-induced MT disassembly (see the tubulin immunostaining) results in a more amoeboid-like phenotype with less spreading on the flat nanolines (left) and nanotextures (right), while developing a strong invasive "in-groove" behavior on the "2.5D" nanotextures (right). Note intact MTs in the control T cells (+DMSO), while Nocodazole-treated T cells display only the diffuse cytoplasmic monomeric tubulin signal. Colors: blue - ICAM1 nanolines (left) or ICAM1-coated nanotexture (right), green - microtubules, red - F-actin, blue - nuclei.

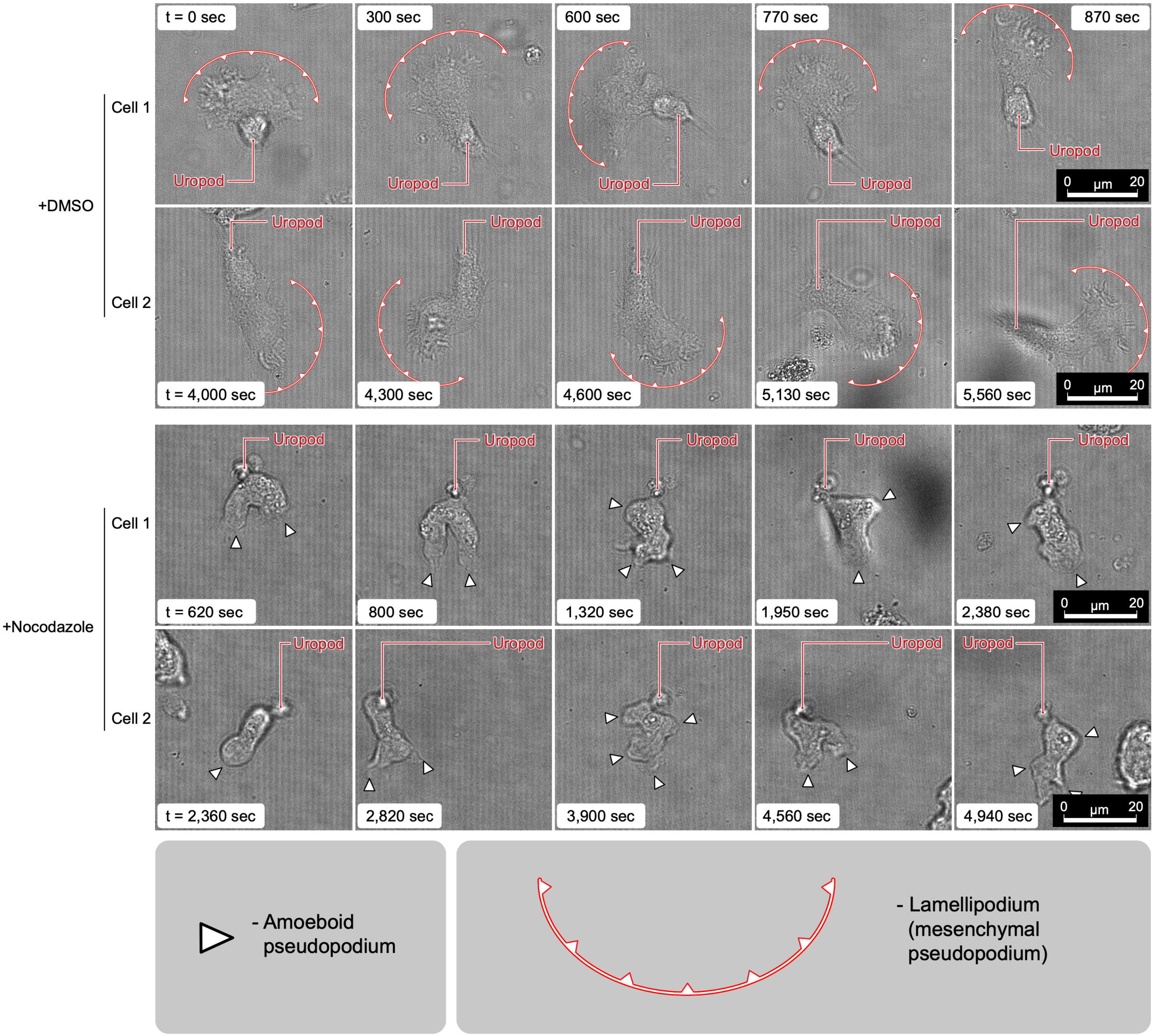

Supplementary Figure 3. Time-course of T cell migration on stiff nanotextured surfaces. Top - +DMSO treatment (control) maintains T cells in the mixed phenotype with well-spread mesenchymal-like “on-ridge” and more amoeboid-like “in-groove” protrusions during migration on stiff (G’=50kPa) nanotextures. We note that the T cells display a strong lamellipodial spreading (comb) and overall cell flattening during the course of cell migration (see also Supplementary Movies 1 and 2). Bottom - Nocodazole treatment induces transition towards a more strongly amoeboid phenotype on stiff nanotextures (see Supplementary Movies 3 and 4) with well-developed pseudopodial amoeboid 3D protrusions (arrowheads).

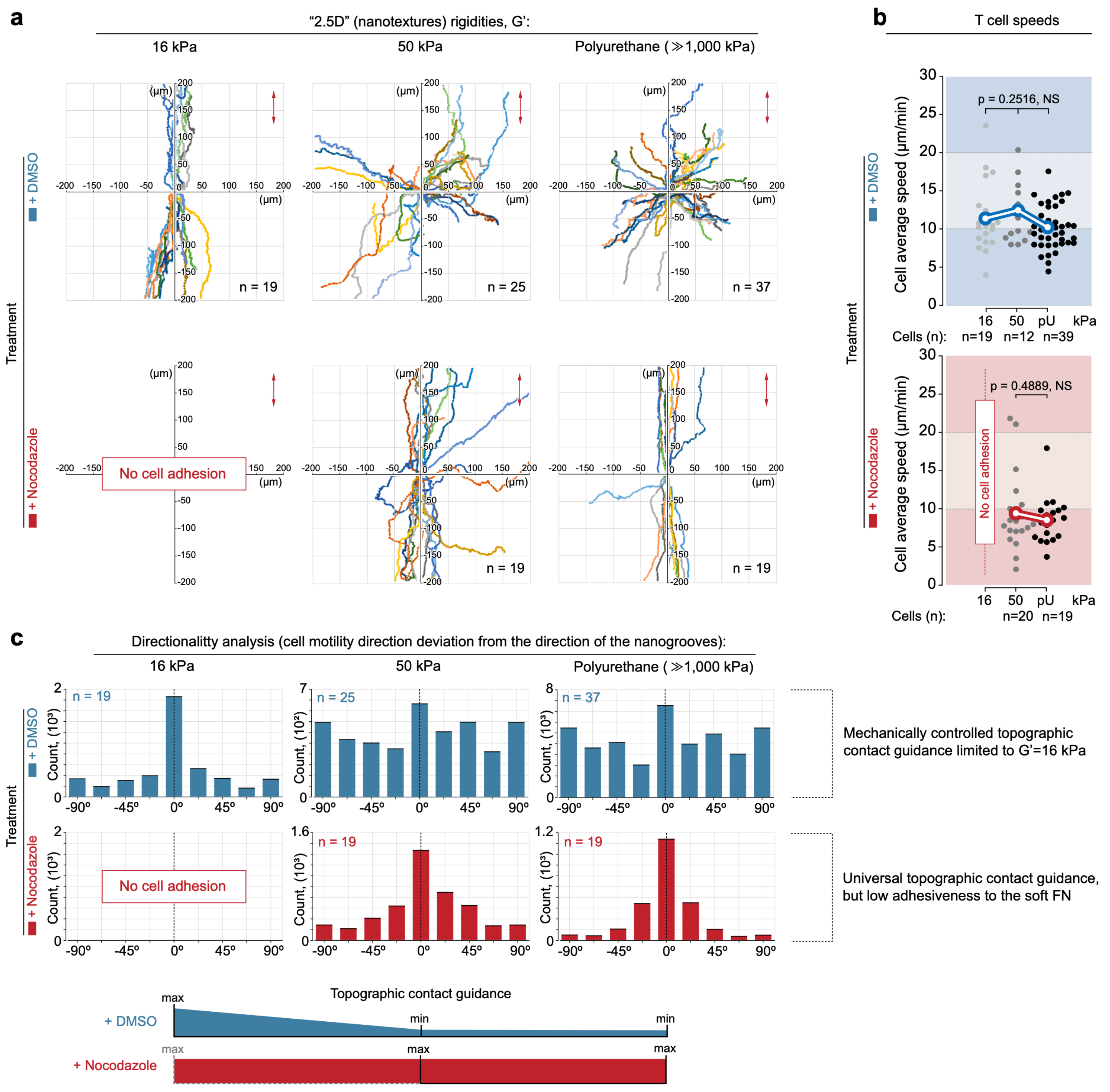

Supplementary Figure 4. T cell migration on fibronectin nanotextures is stiffness- and microtubule-dependent. (a) T cells migration tracks on compliant (G'=16 kPa), intermediate (G'=50 kPa), or rigid (G'≫1,000 kPa) "2.5D" fibronectin (FN) nanotextures, where compliant nanotopographies enhance contact guidance. (Top to bottom) T cell migration under control (+DMSO) or Nocodazole conditions, where disassembly of MTs results in the enhanced directed migration across higher rigidities, but limits adhesion on softer architectures. All n values are shown on the plots. Number of replicates per condition: 2. Source data are provided as a Source Data file.

(b) Average speeds for T cells migrating on FN nanotextures of various rigidities (16, 50 and ≫1,000 kPa) for control (+DMSO, top) and Nocodazole treated conditions. Individual dots correspond to individual cells.

Statistical tests are one-way ANOVA, Tukey's multiple comparisons tests. All n and p values are shown on the plots. Number of replicates per condition: 2. Source data are provided as a Source Data file.

(c) Quantification of contact guidance directionality for human T cells migration on FN nanotextures as a function of substrate mechanical rigidity and microtubule stability. Measurements represent frequency distributions of cell-to-nanogroove angles every 10 s step. All n values are shown on the plots. Number of replicates per condition: 2. Source data are provided as a Source Data file.

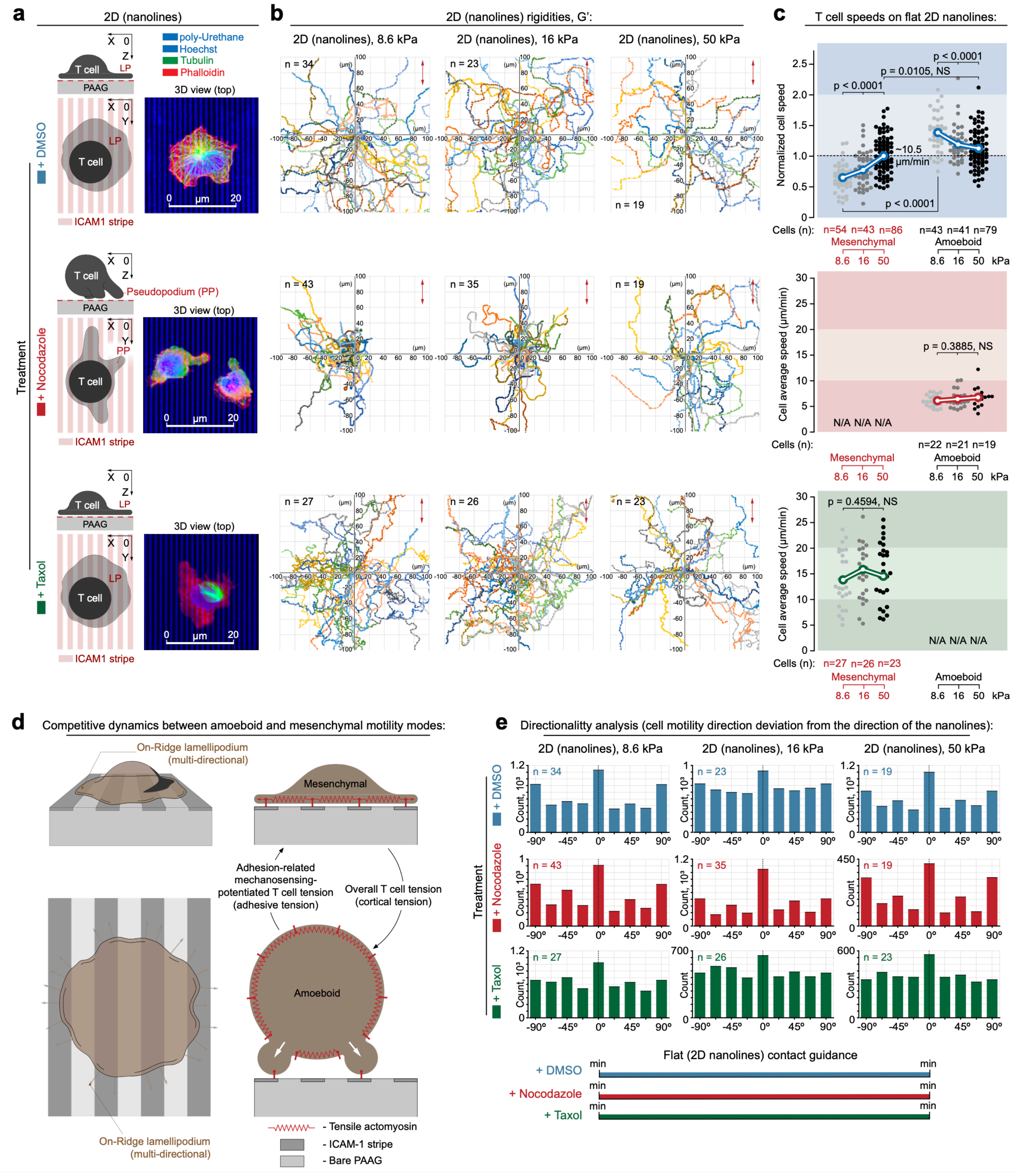

Supplementary Figure 5. The microtubule-dependent amoeboid-mesenchymal phenotype balance influences T cell migration and mechanoresponsive behavior. Using flat anisotropic ICAM1 nanolines, featuring the same lines/gaps dimensionality as “2.5D” nanoridges, i.e., 800 nm-wide lines alternating with 800

nm-wide gaps, we observe T cell behavior that is distinct from behaviors on nanotextured surface and dependent on phenotype.

(a) Left to right: Schematic, 3D micrograph, and cross-section and side views of human T cells (phalloidin - red, nuclei - blue, and tubulin - green) on flat ICAM1 nanolines (blue) (line/gap widths=800 nm). Top to bottom: control (+DMSO), destabilized MTs (+Nocodazole), and stabilized MTs (+Taxol) showing distinct MT stability-dependent phenotypes.

(b) T cell migration on flat lines is largely random with no significant migration alignment to the lines as can be seen from T cells migration tracks on compliant (G'=16 kPa), intermediate (G'=50 kPa), or rigid (G'≫1,000 kPa) flat ICAM1 nanolines. Top to bottom: T cells migration under control (+DMSO), Nocodazole, or Taxol treatment conditions. All n values are shown on the plots. Number of replicates per condition: 4. Source data are provided as a Source Data file.

(c) T cell averaged per cell speeds on ICAM1 nanolines of various rigidities (16, 50 and ≫1,000 kPa) under control (+DMSO, top), Nocodazole, (middle), Taxol (bottom) conditions showing that T cells in the flat control conditions (+DMSO) can display either amoeboid and mesenchymal phenotypes that result in different migration speeds and different mechanoresponsive behaviors, while MT instability shifts T cells to amoeboid behavior and MT stability shifts to the mesenchymal-like behavior. In fact, we observed a range of morphologies and identified coexisting amoeboid-like (more spheroid) and mesenchymal-like (more flattened/spread) phenotypes. Thus, the flat 2D nanolines system allows us to isolate phenotype behaviors that co-exist. As the amoeboid-mesenchymal transition was sporadically frequent, we could not build separate continuous migration tracks over time for each of the cell phenotypic states. However, we quantified migration speed during each phenotypic state and for the mesenchymal phenotype observed increased speed with substrate stiffness (speed goes from 6 to 7.5 to 10 μm/min for G' of 8.6 to 16 to 50 kPa, respectively), consistent with behavior reported for T cells on flat non-patterned ICAM1 surfaces where the distinction between mesenchymal vs. amoeboid T cell phenotypes was not made [24]. In contrast, we defined the opposite trend in speed as a function of substrate stiffness for T cells in the amoeboid phenotype, where average speed decreases from ~15 to ~10 μm/min as nanoline rigidity increases, consistent with observations on nanotextures. Individual dots correspond to individual cells. Statistical tests are one-way ANOVA, Tukey's multiple comparisons tests. All n and p values are shown on the plots. Number of replicates per condition: 4. Source data are provided as a Source Data file.

(d) Left - Schematics summarizing our conclusions for T cell behavior for T cells on flat nanolines showing behavior of more mesenchymal-like (lamellipodial-based) and amoeboid-like phenotypes.

(e) Quantification of directionality of human T cells migration on flat nanolines as a function of substrate mechanical rigidity and the state of microtubules. Corresponding T cells migration tracks are shown in the same matrix order as panel B. Measurements represent frequency distributions of cell-to-nanoline angles every 10 s step. All n values are shown on the plots. Number of replicates per condition: 4. Source data are provided as a Source Data file.

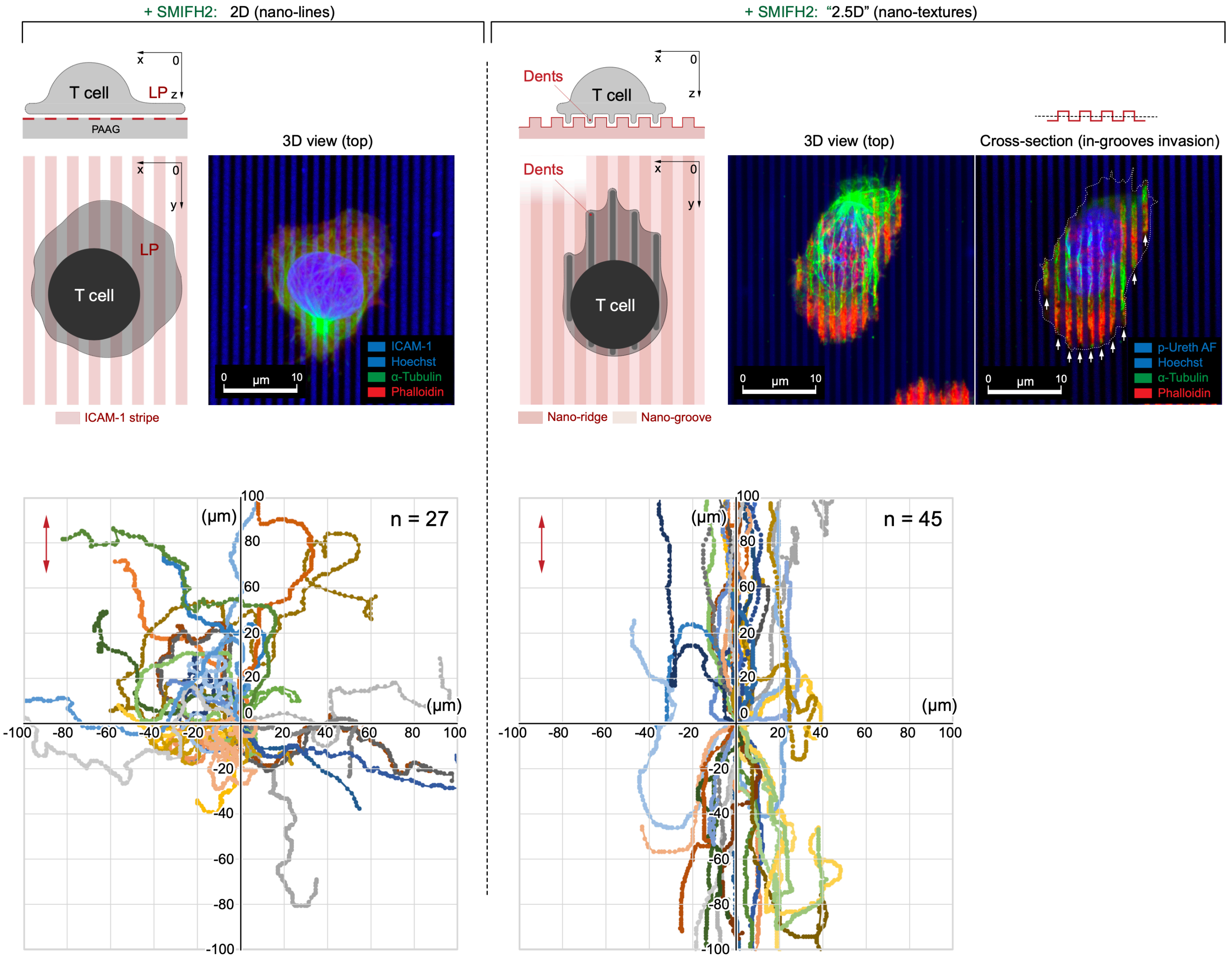

Supplementary Figure 6. T cell morphology, spreading, and "in-groove" invasiveness on stiff substrates under Formins inhibition. Top Left - Flat 2D surface with nanolines . Top Right - Nanotextured "2.5D" surface. Under Formins inhibition, hCD4+ T cells develop a flat spreading architecture on stiff nanolines. On "2.5D" substrates, Formins inhibition shifts the amoeboid-mesenchymal balance more toward the "in-groove" amoeboid-like behavior with more partial "in-groove" invasiveness (right). Bottom Left - T cells migration tracks on flat nanolines showing more random migration. Bottom Right - T cells migration tracks on nanotextures showing highly directed migration, consistent with our hypothesis. Arrows indicate "in-groove" T cell protrusions. Colors: blue - ICAM1 nanolines (left) or ICAM1-coated nanotexture (right), green - microtubules, red - F-actin, blue - nuclei. All n values are shown on the plots. Number of replicates per condition: 2. Source data are provided as a Source Data file.

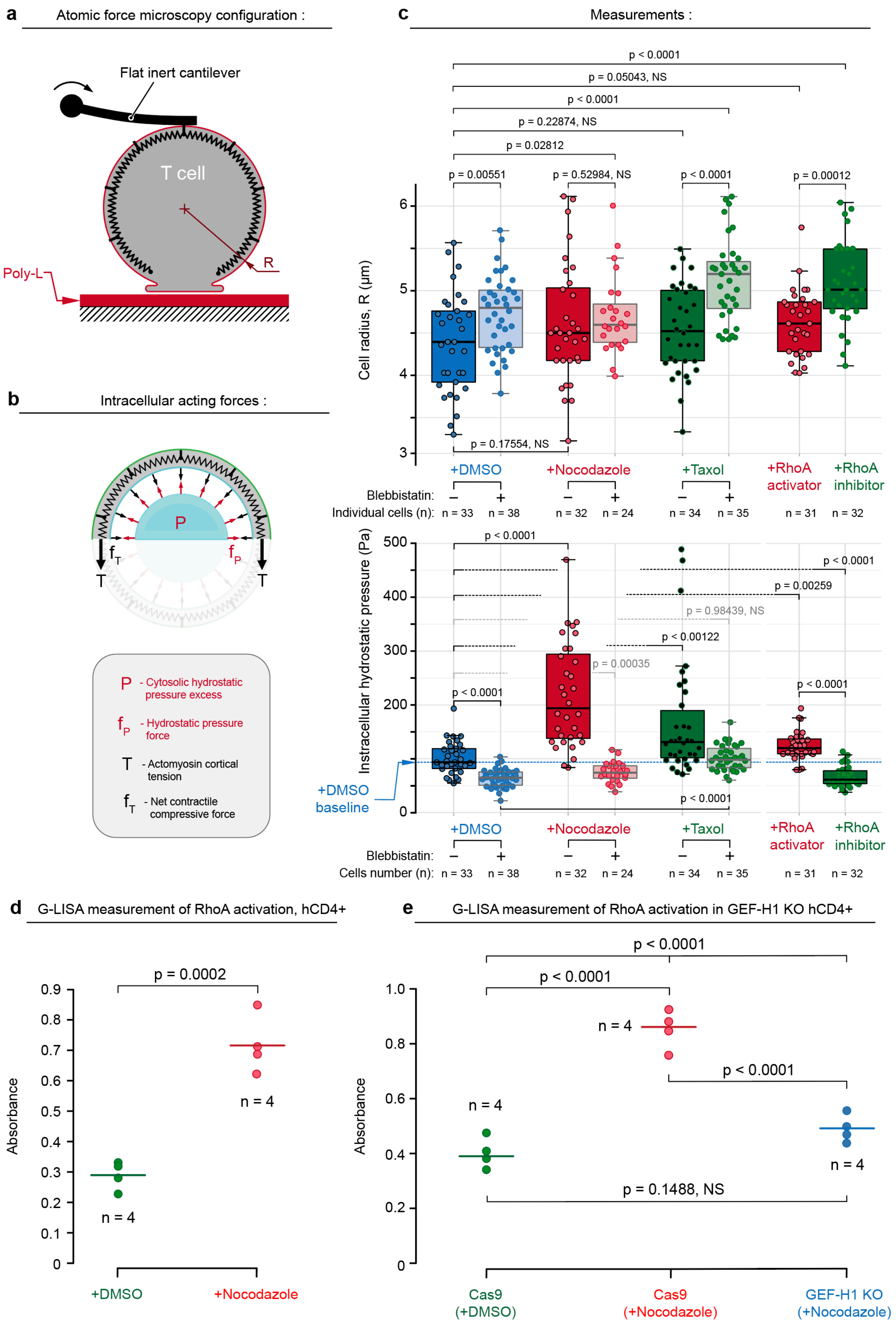

a
Atomic force microscopy configuration :
Flat inert cantilever
T cell
R
Poly-L
b
Intracellular acting forces :
P
f_T
f_P
T
T
P - Cytosolic hydrostatic pressure excess
f_P - Hydrostatic pressure force
T - Actomyosin cortical tension
f_T - Net contractile compressive force
c
Measurements :
p < 0.0001
p = 0.05043, NS
p < 0.0001
p = 0.22874, NS
p = 0.02812
p = 0.00551
p = 0.52984, NS
p < 0.0001
p = 0.00012
Cell radius, R (µm)
6
5
4
3
p = 0.17554, NS
+DMSO
+Nocodazole
+Taxol
+RhoA activator
+RhoA inhibitor
Blebbistatin: − + − + − +
Individual cells (n): n = 33 n = 38 n = 32 n = 24 n = 34 n = 35 n = 31 n = 32
Instracellular hydrostatic pressure (Pa)
500
400
300
200
0
p < 0.0001
p < 0.0001
p = 0.00259
p = 0.98439, NS
p < 0.00122
p = 0.00035
p < 0.0001
p < 0.0001
p < 0.0001
+DMSO baseline
+DMSO
+Nocodazole
+Taxol
+RhoA activator
+RhoA inhibitor
Blebbistatin: − + − + − +
Cells number (n): n = 33 n = 38 n = 32 n = 24 n = 34 n = 35 n = 31 n = 32
d
G-LISA measurement of RhoA activation, hCD4+
p = 0.0002
Absorbance
0.9
0.8
0.7
0.6
0.5
0.4
0.3
0.2
0.1
0
n = 4
n = 4
+DMSO
+Nocodazole
e
G-LISA measurement of RhoA activation in GEF-H1 KO hCD4+
p < 0.0001
p < 0.0001
p < 0.0001
p = 0.1488, NS
Absorbance
1.0
0.8
0.6
0.4
0.2
0
n = 4
n = 4
n = 4
Cas9 (+DMSO)
Cas9 (+Nocodazole)
GEF-H1 KO (+Nocodazole)

Supplementary Figure 7. Intracellular hydrostatic pressure in human T cells.

(a) Schematic diagram of the atomic force microscopy configuration, and **(b)** Schematic diagram illustrating the intracellular force balance in cells under test conditions. The net contractile compressive forces (f_T) generated from the actomyosin cortical tension (*T*) is balanced by the cytosolic hydrostatic pressure excess (*P*). Note that the cortex compressive forces and the corresponding hydrostatic pressure forces (f_P) act in all directions opposite to each other. Additionally, note that the actomyosin cortex is represented as wiggle lines and the incompressible cytosolic fluid in blue.

(c) Individual measurements and calculation of the T cell radii and corresponding hydrostatic pressures: *Top* - Human CD4+ T cell radii distribution with and without pharmacological treatments. *Bottom* - Intracellular hydrostatic "cytoplasmic" pressure derived from T cell surface tension and cell shape. The control group and each of the two MT-targeting treatments (i.e., +DMSO, +Nocodazole and +Taxol, *solid colors*) are paired with blebbistatin co-treatment (*pale semi-transparent colors*) to verify the key role of actomyosin contractility in change of intracellular hydrostatic pressure during MT-targeting. Alternatively, direct RhoA activation or inhibition is compared to MT-perturbation results. Both MT disassembly (+Nocodazole) and direct RhoA activation (+RhoA activator) induce significantly increased cytoplasmic pressure in human T cells via increased actomyosin tension, as demonstrated by AFM findings after blebbistatin co-treatment. MT destabilization or direct RhoA activation induced the rise of hydrostatic pressure. Note that taxol-induced stabilization of MTs increases passive (i.e., actomyosin-independent) resistance to compression via a direct mechanical contribution of stabilized MT scaffolds (+Taxol vs. +Taxol+Blebbistatin treatments). Individual dots correspond to individual cells. Box plots depict the 25th percentile, median, 75th percentile and whiskers depict the 95% confidence intervals. Statistical tests are one-way pairwise t tests. No multiple group comparisons tests are shown. All n and p values are shown on the plots. Number of replicates per condition: 3. Source data are provided as a Source Data file.

(d) Active Rho levels quantified using the G-LISA assay for n=4 control and n=4 Nocodazole treated human T cells. Individual dots correspond to individual experimental replicates. Statistical tests are unpaired t tests. Number of replicates is n and is equal to 4. All n and p values are shown on the plots. Source data are provided as a Source Data file.

(e) Active Rho levels quantified using the G-LISA assay for Cas9 control cells treated with vehicle or Nocodazole and *GEF-H1* KO cells treated with Nocodazole (n=4/condition). Individual dots correspond to separate experimental replicates. Statistical test is a one-way ANOVA with post-hoc Tukey's multiple comparison testing. Number of replicates is n and is equal to 4. All n and p values are shown on the plots. Source data are provided as a Source Data file.

.

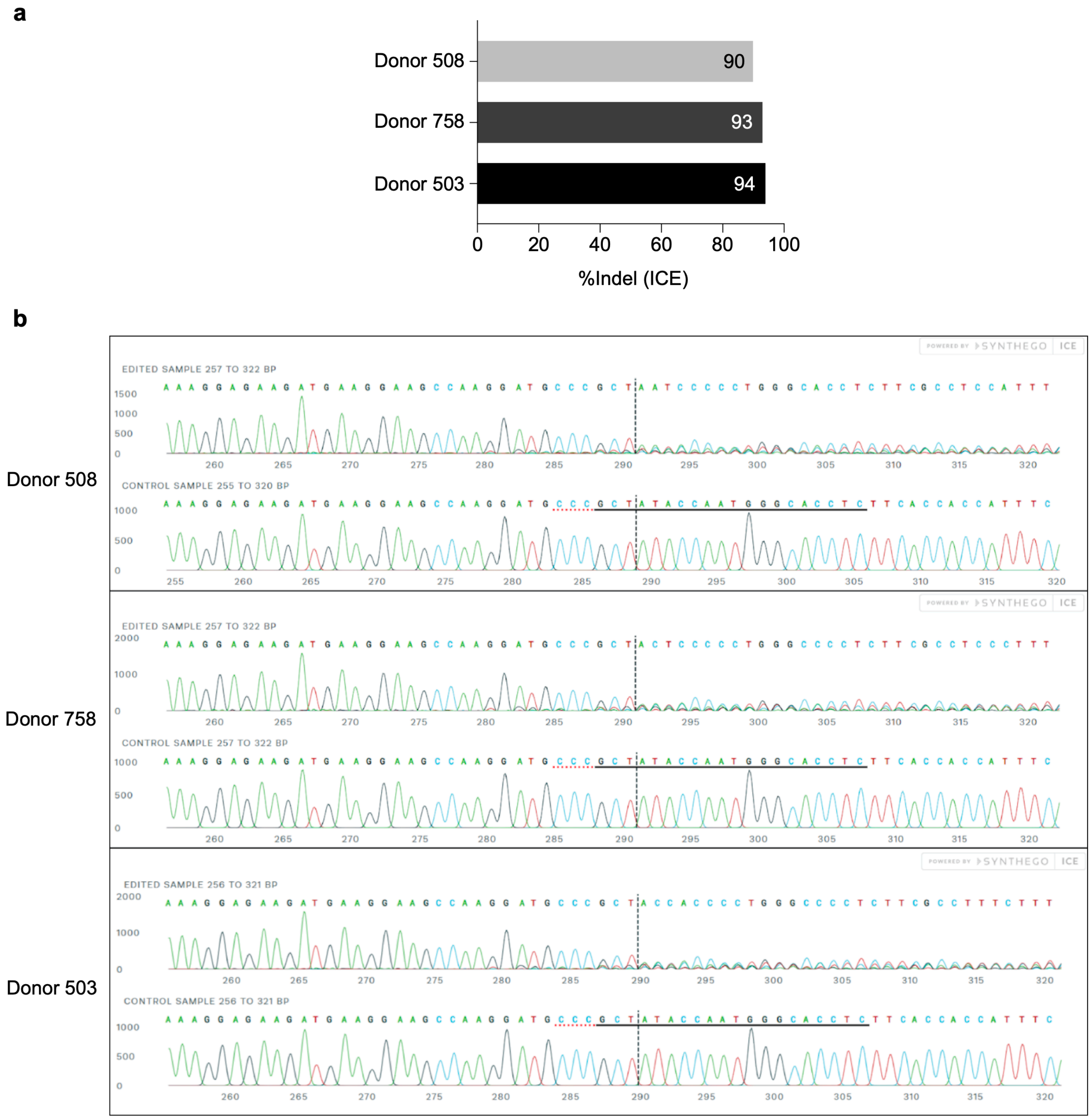

Supplementary Figure 8. Genotyping of *GEF-H1* KO in human CD4+ (hCD4+) T cells. (a) Indel percentage of *GEF-H1* KO in primary T cell lines from 3 separate donors. **(b)** Sanger sequence view showing edited and wild-type (control) sequences in the region around the guide sequence. The hARHGEF2 gRNA sequence is 5'-GAGGTGCCCATTGGTATAGC-3' while the horizontal black underlined region represents the reverse complementary guide sequence. The horizontal red underline is the PAM site while the PAM CRISPR/Cas-9 recognition sequence is GGG. The vertical black dotted line represents the actual cut site.

Supplementary Table 1: Key resources

REAGENT or RESOURCE	SOURCE	IDENTIFIER
**Antibodies**		
AffiniPure Fab Fragment Goat Anti-Human IgG, Fc_{γ} fragment specific	Jackson ImmunoResearch Laboratories, Inc	Cat# 109-007-008; RRID:AB_2632440
Anti-Tubulin antibody [YL1/2]	AbCam	Cat# ab6161; RRID:AB_305329
Anti-Tubulin antibody [SAP.4G5]	Sigma-Aldrich	Cat# T7816, RRID:AB_261770
Atto 647N goat anti-mouse IgG	Sigma-Aldrich	Cat# 50185, RRID:AB_1137661
Alexa Fluor 488 donkey anti-rat IgG (H+L)	Thermo Fisher	Cat# a21208; RRID:AB_141709
**Chemicals, Peptides, and Recombinant Proteins**		
HMS-31, (25-35% Methylhydrosiloxane)-Dimethylsiloxane Copolymer, Trimethylsiloxane Terminated	Gelest, Inc	Cat# HMS-301; CAS#68037-59-2
VDT-731, (7.0-8.0% Vinylmethylsiloxane) - Dimethylsiloxane Copolymer, Trimethylsiloxy Terminated	Gelest, Inc	Cat# VDT-731; CAS#67762-94-1
2,4,6,8-Tetramethyl-2,4,6,8-tetravinylcyclotetrasiloxane	Sigma-Aldrich	Cat# 396281; CAS#2554-06-5
Platinum(0)-2,4,6,8-tetramethyl-2,4,6,8-tetravinylcyclotetrasiloxane complex solution	Sigma-Aldrich	Cat# 479543; CAS#68585-32-0
SYLGARD 184 Silicone Elastomer Kit, 0.5 kg KIT	Dow Corning, Sigma-Aldrich	Cat# 4019862; CAS#68988-89-6
40% Acrylamide Solution, Electrophoresis purity reagent, 500mL	BioRad	Cat# 161-0140
2% Bis Solution, 500mL	BioRad	Cat# 161-0142
Streptavidin Acrylamide, 1 mg	Thermo Fisher, Life Technologies	Cat# S21379
TEMED	Thermo Scientific	Cat# 17919; CAS#110-18-9
Ammonium Persulfate, BioUltra, for molecular biology	Fluka Analytical	Cat# 09913-100G; CAS#7727-54-0
3-(Trimethoxysilyl)propyl methacrylate	Sigma-Aldrich	Cat# 6514; CAS#2530-85-0
Ethyl Alcohol 200 Proof, Absolute, Anhydrous ACS/USP Grade	Pharmco-Aaper	Cat# 111000200; CAS#64-17-5

Collagen Type I, High Concentration, Rat Tail, 100mg	VWR	Cat# 47747-218
Fibronectin Bovine Protein, Plasma	Thermo Fisher, Life Technologies	Cat# 33010018
Genemate LE Quick DIssolve Agarose	BioExpress	Cat# E-3120-500FG
ICAM1, Human Protein, Recombinant, hIgG1-Fc.His Tag, Active	Sino Biological, China	Cat# 10346-H03H-50
Paraformaldehyde, reagent grade, crystalline	Sigma-Aldrich	Cat# P6148-500G; CAS#30525-89-4
Methanol, HPLC pure, >99.9%	Sigma-Aldrich	Cat# 34860-4L-R; CAS#67-56-1
Triton X-100	Sigma-Aldrich (Roche)	Cat# 11332481001 CAS#9002-93-1
(−)-Blebbistatin, 1-Phenyl-1,2,3,4-tetrahydro-4-hydroxypyrrolo[2.3-b]-7-methylquinolin-4-one	Sigma-Aldrich	Cat# 203391; CAS#856925-71-8
Nocodazole, Microtubule Inhibitor	AbCam	Cat# ab120630; CAS#31430-18-9
Paclitaxel (Taxol)	Sigma-Aldrich	Cat# T7402; CAS#33069-62-4
0.25% Trypsin, 2.21 mM EDTA, 1× [-] sodium bicarbonate	Corning	Cat# 25-053-CI
Plasmocin	Fisher Scientific	Cat# NC9698402
Penicillin Streptomycin Solution, 100X	Corning	Cat# 30-002-CI
Fetal Bovine Serum	HyClone	Cat# SH30910.03
Acetic Acid, Glacial	Fisher Chemical	Cat# BP2401-500; CAS#64-19-7
DMSO (Dimethyl sulfoxide)	Sigma-Aldrich	Cat# 472301-100ML; CAS#67-68-5
Silanization solution-I	Sigma-Aldrich	Cat# 85126; CAS#75-78-5
35 mm NanoSurface Dish	NanoSurface Biomedical, Seattle, WA	Cat# ANFS-0001
25 mm NanoSurface Coverglass	NanoSurface Biomedical, Seattle, WA	Cat# ANFS-CS25
35 mm Dish | No. 1.0 Coverslip | 20 mm Glass Diameter | Uncoated	MatTek Corporation	Cat# P35G-1.0-20-C
Cover Glasses, FisherFinest Rectangles, 25×60-1, Thickness 0.13-0.17 mm	Fisher Scientific	Cat#24X60-1
Cover Glasses, Circles, 15 mm, Thickness 0.13-0.17 mm	Carolina Biological Supply Company	Cat# 633031
CellTracker Green CMFDA (5-chloromethylfluorescein diacetate)	Thermo Fisher Scientific	Cat# C7025

CellTracker Red CMTPX	Thermo Fisher Scientific	Cat# C34552
SiR Actin, Spirochrome Kit	Cytoskeleton Inc.	Cat# CY-SC001; CAS#1640283-36-8
Bovine Serum Albumin (BSA), fatty acid-free powder	Fisher Bioreagents	Cat# BP9704-100; CAS#9048-46-8
Phalloidin-iFluor 647 Reagent - CytoPainter	AbCam	Cat# ab176759
(+)-Biotin N-hydroxysuccinimide ester	Sigma-Aldrich	Cat# H1759; CAS#35013-72-0
Alexa Fluor 568 carboxic acid, succinimidyl ester	Molecular Probes	Cat# A20003
Slide-A-Lyzer MINI Dialysis Device, 7K MWCO, 0.1 mL	Thermo Fisher	Cat# 69560
Rho Activator II	Cytoskeleton, Inc.	Cat# CN03-A
Rho Inhibitor I	Cytoskeleton, Inc.	Cat# CT04-A
Soybean Trypsin Inhibitor	ATCC	Cat# 30-2104
Hepes (1M)	Thermo Fisher Scientific	Cat# 15630080
Leibovitz's L-15 Medium	Thermo Fisher Scientific	Cat# 11415064
Phosphate Buffered Saline (PBS) pH 7.4 (1X)	Thermo Fisher Scientific	Cat# 10010023
Penicillin Streptomycin Solution, 100X	Corning	Cat# 30-002-CI
DMEM, 1X (Dulbecco's Modification of Eagle's Medium) with4.5 g/L glucose, L-glutamine & sodium pyruvate	Corning	Cat# 10-013-CV
RPMI 1640 with L-glutamine	Corning	Cat# 10-040-CV
EasySep Human CD4+ T Cell Isolation Kit	STEMCELL Technologies, Inc., USA	Cat# 17952
EasySep Mouse CD8+ T Cell Isolation Kit	STEMCELL Technologies, Inc., USA	Cat# 19853
ImmunoCult Human CD3/CD28/CD2 T Cell Activator	STEMCELL Technologies Inc., USA	Cat# 10970
Dynabeads Mouse T-Activator CD3/CD28	Thermo Fisher Scientific	Cat# 11456D
"The Big Easy" EasySep Magnet	STEMCELL Technologies, Inc., USA	Cat# 18001
MojoSort Human CD4 Nanobeads	BioLegend	Cat# 480014

GE Healthcare Ficoll-Paque PLUS Media	Thermo Fisher Scientific	Cat# 45-001-750
Ammonium-Chloride-Potassium (ACK) Lysing Buffer	VWR International	Cat# 118-156-101
Dynabeads Human T-Activator CD3/CD28 for T Cell Expansion and Activation	Gibco	Cat# 11132D
CTS Immune Cell SR	Gibco	Cat# A2596101
Human K562	ATCC	Cat# CCL-243
Recombinant Human IL-2 (rhIL-2)	PeproTech	Cat# 200-02
Recombinant human IL-7 (rhIL-7)	PeproTech	Cat# 200-07
Recombinant human IL-15 (rhIL-15)	STEMCELL Technologies, Inc., USA	Cat# 78031
CTS OpTmizer T Cell Expansion SFM	Gibco	Cat# A1048501
L-glutamine (200mM)	Gibco	Cat# 25-030-081
EmbryoMax Penicillin-Streptomycin Solution, 100X	Sigma-Aldrich	Cat# TMS-AB2-C
ImmunoCult-XF T Cell Expansion Medium	STEMCELL Technologies, Inc., USA	Cat# 10981
ImmunoCult Human CD3/CD28/CD2 T Cell Activator	STEMCELL Technologies, Inc., USA	Cat# 10990
Human Recombinant IL-2, ACF	STEMCELL Technologies, Inc., USA	Cat# 78145.1
0.01% poly-L-lysine	Sigma-Aldrich	Cat# P4707; CAS#25104-18-1
**Experimental Models: Cell Lines**		
Human CD4+ T cells (derived from commercially supplied human whole peripheral blood).	STEMCELL Technologies, Inc., USA	Cat#70507.1
Human CD4+ T cells (derived from human whole peripheral blood).	Memorial Blood Centers, Minneapolis, MN	Primary cells
Mouse CD8+ T cells (derived from mouse spleen).	*KPC(T)* mouse, UMN	Primary cells
**Experimental Models: Organisms/Strains**		
KPC(T) Mouse line	NCI Mouse Repository or Jackson Labs	All mice bred at the University of MInnesota. Mice were originally obtained from either the NCI mouse repository under Strain Numbers

		01XJ6 (*Kras*$^{LSL\text{-}G12D/+}$), 01XM2 (*p53*$^{LSL\text{-}R172H/+}$), and 01XL5 (*Pdx1-Cre)* or Jackson Labs for tdTomato mice (Stock Number 007914).
**Oligonucleotides**		
sgRNA targeting human ARHGEF2: 5'-GAGGTGCCCATTGGTATAGC-3'	Synthego	N/A
CleanCap Cas9 mRNA	TriLink Biotechnologies	Cat# L-7606
**Software and Algorithms**		
NIS-Elements Advanced Research 3.0	Nikon Instruments	RRID:SCR_014329
NIS-Elements Confocal software 3.0	Nikon Instruments	RRID:SCR_002776
KaleidaGraph 4.5.3	http://www.synergy.com/	RRID:SCR_014980
GraphPad Prism 7b	https://www.graphpad.com/	RRID:SCR_002798
PlotsOfData	https://huygens.science.uva.nl	N/A
Adobe Illustrator CC, 21.0.0.	Adobe Systems, Inc.	RRID:SCR_010279
Huygens Professional software version 18.10.0	SVI, Hilversum, NL https://svi.nl/Huygens-Professional	
NanoScope Analysis 1.7	Bruker	
MATLAB R2019a	The MathWorks	
**Other**		
AFM Tipless Probe	MikroMasch (μMasch), Germany	Cat# HQ:CSC38/tipless/Cr-Au
Leica SP8 STED 3X system	Leica Microsystems https://www.leica-microsystems.com/	
iSIM microscopy system	VisiTech Intl., Sunderland, UK	
Nikon A1Rsi Confocal w/ SIM Super Resolution	Nikon Instruments https://www.microscope.healthcare.nikon.com/products/confocal-microscopes/a1hd25-a1rhd25	

MATLAB Code

```
clear all
clc
nooftimepts=30; % (# of frames) change depending on frequency of intervals
totcount=0;
cellcount=0;
timeinterval= 1.5; % (1.5min/frame) change depending on frequency of interval
str1='Tracks_s';
str3='.xml';
% str4='drift_s';
a_1=[3,4];
dev=[1.5,4];

for q=1:1
    str2=num2str(a_1(q));
    path=strcat(str1,str2,str3);

    [tracks, md] = importTrackMateTracks('');  %% importing tracks from a
trackmate file

%  tracks(#) = []; DELETE ME I AM FOR REMOVING A BAD TRACK

for n=1:size(tracks,1) %%number of tracks
    Track=tracks{n,1};  %% EXTRACTING EACH TRACK: no of spots x 4; Columns denote
serial numbers, x, y, z.
    for m=1:size(Track,1) %% no of timepoints for a particular track
        Xii(m+totcount,1)=Track(m,2)*1.164; %% putting all of the x values in a
column vector of size nooftimepts*noofcells
        Yii(m+totcount,1)=Track(m,3)*1.164; %% 1.164 is converting pixel output
of trackmate to micron
        Zii(m+totcount,1)=Track(m,4)*5;     %% 5 is setting the z-step size (5um)
between images in the stack
    end
    noofspots(n+cellcount)=m;
    totcount=totcount+nooftimepts;
end
    cellcount=cellcount+n;
    trackcount(q)=cellcount;
end

Xi=zeros(totcount,1);
Yi=zeros(totcount,1);
Zi=zeros(totcount,1);


for k=1:size(Xii,1)
    Xi(k,1)=Xii(k,1);
    Yi(k,1)=Yii(k,1);
    Zi(k,1)=Zii(k,1);
end                     %%accounting for the incomplete last cell track so that
everything is in order for the subsequent analysis


noofcells=size(Xi,1)/nooftimepts;
trajx=transpose(reshape(Xi,[nooftimepts,noofcells]));
```

```
trajy=transpose(reshape(Yi,[nooftimepts,noofcells]));
trajz=transpose(reshape(Zi,[nooftimepts,noofcells]));

XX=zeros(noofcells, nooftimepts);
YY=zeros(noofcells, nooftimepts);
ZZ=zeros(noofcells, nooftimepts);

for i=1:noofcells
    for j=1:noofspots(1,i)-1
       %traject_x_1 & traject_y_1 are similar to other analyses
        if j==noofspots(1,i)
            continue
        else
            traject_x_1(i,j)=(trajx(i,j+1)-trajx(i,j));
            traject_y_1(i,j)=(trajy(i,j+1)-trajy(i,j));
            traject_z_1(i,j)=(trajz(i,j+1)-trajz(i,j));
        end
        %% Correction code use only if needed
        [theta,rho]=cart2pol(traject_x_1(i,j),traject_y_1(i,j)); %% shifting the
axes x and y so that x is along the median fiber alignment angle and y orthogonal
to it
        theta=(theta+dev(1+floor(i/trackcount(q)))*0*pi/180); % average deviation
of principal fiber orientation axis captured in "dev" for each field of view from
CT FIRE analysis
        [xx,yy]=pol2cart(theta, rho);
        traject_x_1(i,j)=xx;
        traject_y_1(i,j)=yy;
    %%
%          XX(i,j+1)=traject_x_1(i,j)+XX(i,j)-drift(j,1);
%          YY(i,j+1)=traject_y_1(i,j)+YY(i,j)-drift(j,2);
%          ZZ(i,j+1)=traject_z_1(i,j)+ZZ(i,j)+drift(j,3);
           XX(i,j+1)=traject_x_1(i,j)+XX(i,j);
           YY(i,j+1)=traject_y_1(i,j)+YY(i,j);
           ZZ(i,j+1)=traject_z_1(i,j)+ZZ(i,j);
%          ZZ(i,j+1)=traject_z_1(i,j)+ZZ(i,j);
    end
%     plot(XX(i,:),YY(i,:),'Color',[0,0.5,0])

plot3(XX(i,1:min(noofspots(1,i),25)),YY(i,1:min(noofspots(1,i),25)),ZZ(i,1:min(no
ofspots(1,i),25)),'r')
%     plot3(XX(i,:),YY(i,:),ZZ(i,:),'k')
    hold on

end

axis equal
% axis([-100 100 -100 100])
dist_x=abs(traject_x_1);
dist_y=abs(traject_y_1);
dist_z=abs(traject_z_1);

for i=1:noofcells
totdist_x(i)=sum(dist_x(i,:));
totdist_y(i)=sum(dist_y(i,:));
totdist_z(i)=sum(dist_z(i,:));
end
```

```
avg_speed_x=(60/timeinterval)*totdist_x./(noofspots-1); %% ballistic avg speed in
um/h
avg_speed_y=(60/timeinterval)*totdist_y./(noofspots-1);
avg_speed_z=(60/timeinterval)*totdist_z./(noofspots-1);

%% Fit PRWModel
    [speed_x, pers_x, R_pers_x]=PRWM_3D(XX, noofspots); %R_pers_x is the R-
squared value for the non linear regression
    [speed_y, pers_y, R_pers_y]=PRWM_3D(YY, noofspots);
    [speed_z, pers_z, R_pers_z]=PRWM_3D(ZZ, noofspots);

%% Weed out tracks
speed=[speed_x speed_y speed_z];
pers=[pers_x pers_y pers_z];
R=[R_pers_x R_pers_y R_pers_z];

meanR=(R_pers_x+R_pers_y+R_pers_z)./3;
q=0;
h=figure;
%figure(1);

for p=1:size(speed_x,1)
    if meanR(p)>0.5 && abs(pers(p,1))<1 && abs(pers(p,2))<1 && abs(pers(p,3))<1
       q=q+1;

plot3(XX(p,1:min(noofspots(1,p),25)),YY(p,1:min(noofspots(1,p),25)),ZZ(p,1:min(no
ofspots(1,p),25)),'g')
       hold on
       speed_corr(q,:)=speed(p,:); % um/h
       pers_corr(q,:)=pers(p,:); % h
       motility(q,:)=speed_corr(q,:).^2.*pers_corr(q,:); % um^2/h
       tot_motility(q,1)=sum(motility(q,:)); % um^2/h
       aniso_x(q,1)=motility(q,1)/tot_motility(q);
       aniso_y(q,1)=motility(q,2)/tot_motility(q);
       aniso_z(q,1)=motility(q,3)/tot_motility(q);
    end
end
axis equal
axesLabelsAlign3D();
xlabel('X (�m)')
ylabel('Y (�m)')
zlabel('Z (�m)')
% xlim([-150 150]);
% ylim([-150 150]);
% zlim([-20 20]);
ax = gca;
ax.BoxStyle = 'full';
box(ax,'on')

%hold off;
%saveas(h,figure_name,'png')

speedsquared=speed_corr.^2;
S=sum(speedsquared,2);
root3Dspeed=sqrt(S); % um/h
```
