# Supplementary material for: Engineering T cells to enhance 3D migration through structurally and mechanically complex tumor microenvironments
Source: Nat Commun. 2021 May 14;12:2815. doi: 10.1038/s41467-021-22985-5 (PMC8121808; doi:10.1038/s41467-021-22985-5)
Supplement: Supplementary file 3 — Description of Additional Supplementary Files [file 41467_2021_22985_MOESM3_ESM.pdf]

Description of Additional Supplementary Files

File Name: Supplementary Movie 1
Description: T cell migration on 50kPa nanogrooves under control conditions. (Left) Low magnification view showing population level T cell migration showing limited directed motility. (Right) Higher magnification view of migrating T cells showing “on-ridge” lamellipodia.

File Name: Supplementary Movie 2
Description: High magnification movie of T cells migration on 50kPa nanogrooves under control conditions. (Left) Lower magnification view. (Middle and Right) High magnification movies of migrating T cells T cells showing “on-ridge” lamellipodia.

File Name: Supplementary Movie 3
Description: T cell migration on 50kPa nanogrooves in the presence of Nocodazole. (Left) Low magnification view showing population level T cell migration showing highly directed motility. (Right) Higher magnification view of migrating T cells with a more “in-groove” dominant (lacking significant lamellipodia) more amoeboid phenotype.

File Name: Supplementary Movie 4
Description: High magnification movie of T cells migration on 50kPa nanogrooves in the presence of Nocodazole. (Left) Lower magnification view. (Middle and Right) High magnification movies of migrating T cells showing highly directed migration with the “in-groove” dominant (lacking significant “on-ridge” lamellipodia) more amoeboid phenotype.
