# Supplementary material for: Engineering T cells to enhance 3D migration through structurally and mechanically complex tumor microenvironments
Source: Nat Commun. 2021 May 14;12:2815. doi: 10.1038/s41467-021-22985-5 (PMC8121808; doi:10.1038/s41467-021-22985-5)
Supplement: Supplementary file 8 — Reporting Summary [file 41467_2021_22985_MOESM8_ESM.pdf]

nature research

Corresponding author(s): Paolo Provenzano

Last updated by author(s): February 2021

# Reporting Summary

Nature Research wishes to improve the reproducibility of the work that we publish. This form provides structure for consistency and transparency in reporting. For further information on Nature Research policies, see our Editorial Policies and the Editorial Policy Checklist.

## Statistics

For all statistical analyses, confirm that the following items are present in the figure legend, table legend, main text, or Methods section.

| n/a | Confirmed | |
|---|---|---|
| ☐ | ☒ | The exact sample size ($n$) for each experimental group/condition, given as a discrete number and unit of measurement |
| ☐ | ☒ | A statement on whether measurements were taken from distinct samples or whether the same sample was measured repeatedly |
| ☐ | ☒ | The statistical test(s) used AND whether they are one- or two-sided<br>*Only common tests should be described solely by name; describe more complex techniques in the Methods section.* |
| ☒ | ☐ | A description of all covariates tested |
| ☐ | ☒ | A description of any assumptions or corrections, such as tests of normality and adjustment for multiple comparisons |
| ☐ | ☒ | A full description of the statistical parameters including central tendency (e.g. means) or other basic estimates (e.g. regression coefficient) AND variation (e.g. standard deviation) or associated estimates of uncertainty (e.g. confidence intervals) |
| ☐ | ☒ | For null hypothesis testing, the test statistic (e.g. $F$, $t$, $r$) with confidence intervals, effect sizes, degrees of freedom and $P$ value noted<br>*Give P values as exact values whenever suitable.* |
| ☒ | ☐ | For Bayesian analysis, information on the choice of priors and Markov chain Monte Carlo settings |
| ☒ | ☐ | For hierarchical and complex designs, identification of the appropriate level for tests and full reporting of outcomes |
| ☒ | ☐ | Estimates of effect sizes (e.g. Cohen's $d$, Pearson's $r$), indicating how they were calculated |

*Our web collection on statistics for biologists contains articles on many of the points above.*

## Software and code

Policy information about availability of computer code

| | |
|---|---|
| Data collection | All software with key information is listed in the Key Resources Table in the Methods Section:<br><br>Software and Algorithms<br>Leica Application Suite LAS X Life Science imaging software, (V. 2020). RRID:SCR_013673<br>NIS-Elements Advanced Research 3.0 Nikon Instruments RRID:SCR_014329<br>NIS-Elements Confocal software 3.0 Nikon Instruments RRID:SCR_002776<br>NanoScope Analysis 1.7 Bruker |
| Data analysis | All software with key information is listed in the Key Resources Table in the Methods Section:<br><br>Software and Algorithms<br>Leica Application Suite LAS X Life Science imaging software, (V. 2020). RRID:SCR_013673<br>NIS-Elements Advanced Research 3.0 Nikon Instruments RRID:SCR_014329<br>NIS-Elements Confocal software 3.0 Nikon Instruments RRID:SCR_002776<br>Fiji, 2.0.0-rc-69/1.52q<br>KaleidaGraph 4.5.3 http://www.synergy.com/ RRID:SCR_014980<br>GraphPad Prism, versions 7b and 8.1.2 https://www.graphpad.com/ RRID:SCR_002798<br>PlotsOfData https://huygens.science.uva.nl N/A<br>Adobe Illustrator CC, 21.0.0. Adobe Systems, Inc. RRID:SCR_010279<br>Huygens Professional software version18.10.0 SVI, Hilversum, NL https://svi.nl/Huygens-Professional<br>NanoScope Analysis 1.7 Bruker |

MATLAB R2019a The MathWorks

For manuscripts utilizing custom algorithms or software that are central to the research but not yet described in published literature, software must be made available to editors and reviewers. We strongly encourage code deposition in a community repository (e.g. GitHub). See the Nature Research guidelines for submitting code & software for further information.

## Data

Policy information about availability of data

All manuscripts must include a data availability statement. This statement should provide the following information, where applicable:

- Accession codes, unique identifiers, or web links for publicly available datasets
- A list of figures that have associated raw data
- A description of any restrictions on data availability

The authors declare that all data supporting the findings of this study are available within the paper and its Supplementary Information Files or from the authors upon reasonable requests.  A complete Data Availability Statement has been added to the manuscript as well a Source Data File.

# Field-specific reporting

Please select the one below that is the best fit for your research. If you are not sure, read the appropriate sections before making your selection.

[x] Life sciences    [ ] Behavioural & social sciences    [ ] Ecological, evolutionary & environmental sciences

For a reference copy of the document with all sections, see nature.com/documents/nr-reporting-summary-flat.pdf

# Life sciences study design

All studies must disclose on these points even when the disclosure is negative.

| | |
|---|---|
| Sample size | Sample sizes varied from ~10 through over 500, depending on the availability of larger sample sets and inherent to each method technical limitations. |
| Data exclusions | No data was excluded. |
| Replication | Several replicates (independent experiments) were conducted for each data set. Number of successful replicates were at least 3. |
| Randomization | Randomization was ensured by the blind picking of the regions of the interest (ROI), e.g. the microscopy of the samples was performed following the  ROI choice across all samples and experiments after defining the true geometric centre of the sample.   Likewise, randomization was used for the choice of the region of the interest (ROI), within each ROI the entire cell population was measured, as long as cells were attached and developed adhesion and a spreading-relevant phenotype. |
| Blinding | Sample blinding is not relevant to this study design. |

# Reporting for specific materials, systems and methods

We require information from authors about some types of materials, experimental systems and methods used in many studies. Here, indicate whether each material, system or method listed is relevant to your study. If you are not sure if a list item applies to your research, read the appropriate section before selecting a response.

## Materials & experimental systems

| n/a | Involved in the study | |
|---|---|---|
| [ ] | [x] | Antibodies |
| [ ] | [x] | Eukaryotic cell lines |
| [x] | [ ] | Palaeontology and archaeology |
| [ ] | [x] | Animals and other organisms |
| [x] | [ ] | Human research participants |
| [x] | [ ] | Clinical data |
| [x] | [ ] | Dual use research of concern |

## Methods

| n/a | Involved in the study | |
|---|---|---|
| [x] | [ ] | ChIP-seq |
| [x] | [ ] | Flow cytometry |
| [x] | [ ] | MRI-based neuroimaging |

## Antibodies

| | |
|---|---|
| Antibodies used | All antibodies used are listed in the Key Resources Table in the Methods Section:<br><br>AffiniPure Fab Fragment Goat Anti-Human IgG, Fcγ fragment specific Jackson ImmunoResearch Laboratories, Inc<br>Cat# 109-007-008; RRID:AB_2632440 |

| | |
|---|---|
| | Anti-Tubulin antibody [YL1/2] AbCam Cat# ab6161; RRID:AB_305329<br><br>Alexa Fluor 488 donkey anti-rat IgG (H+L) Thermo Fisher Cat# a21208; RRID:AB_141709 |
| Validation | AffiniPure Fab Fragment Goat Anti-Human IgG, Fcγ fragment specific Jackson ImmunoResearch Laboratories, Inc Cat# 109-007-008; RRID:AB_2632440 validated by showing by immunoelectrophoresis and/or ELISA that the antibody reacts with the Fc portion of human IgG heavy chain but not with the Fab portion of human IgG. https://www.jacksonimmuno.com/catalog/products/109-007-008<br><br>α-Tubulin rat mAb (AbCam) commercially tested for IHC-Fr, IP, RIA, Western Blot, Flow Cytometry, ICC/IF, IHC (PFA), IHC-P, IHC-Wholempunt, IHC (Methanol, see PubMed:16943269). See https://www.abcam.com/tubulin-antibody-yl12-loading-control-ab6160.html<br><br>Alexa Fluor 488 donkey anti-rat IgG (H+L) Thermo Fisher Cat# a21208; RRID:AB_141709 validated by IHC, IF, and ICC, see https://www.thermofisher.com/antibody/product/Donkey-anti-Rat-IgG-H-L-Highly-Cross-Adsorbed-Secondary-Antibody-Polyclonal/A-21208 |

## Eukaryotic cell lines

Policy information about cell lines

| | |
|---|---|
| Cell line source(s) | Primary murine T cells isolated from KPC mice and primary human T cells from human blood via StemCell or the Minnesota Memorial Blood Centers. |
| Authentication | Blood tested to by HIV, Hep C negative, positive for T cell markers, and functional in response to T cell activators. |
| Mycoplasma contamination | Cell line was continuously monitored for Mycoplasma. |
| Commonly misidentified lines (See ICLAC register) | None of the commonly misidentified cell lines was used in this study. |

## Animals and other organisms

Policy information about studies involving animals; ARRIVE guidelines recommended for reporting animal research

| | |
|---|---|
| Laboratory animals | Genetically engineered Murine Models were used in this study on the mixed or C57/Bl6 background. We have also added additional information to the Supplementary Table (All mice bred at the University of MInnesota. Mice were originally obtained from either the NCI mouse repository under Strain Numbers 01XJ6 (KrasLSL-G12D/+), 01XM2 (p53LSL-R172H/+), and 01XL5 (Pdx1-Cre) or Jackson Labs for tdTomato mice (Stock Number 007914). We have added additional data to the Methods and Supp. Table to describe the mice and their housing. |
| Wild animals | No wild animals were used in the study. |
| Field-collected samples | No Field-collected samples were used in the study. |
| Ethics oversight | All animal studies were approved by the Institutional Animal Care and Use Committee of the University of Minnesota. |

Note that full information on the approval of the study protocol must also be provided in the manuscript.
